# Impact of aging on gene expression response to x-ray irradiation using mouse blood

**DOI:** 10.1038/s41598-021-89682-7

**Published:** 2021-05-13

**Authors:** Constantinos G. Broustas, Axel J. Duval, Sally A. Amundson

**Affiliations:** Center for Radiological Research, Columbia University Vagelos College of Physicians and Surgeons, Columbia University Irving Medical Center, 630 W. 168th St., New York, NY 10032 USA

**Keywords:** Computational biology and bioinformatics, Molecular biology

## Abstract

As a radiation biodosimetry tool, gene expression profiling is being developed using mouse and human peripheral blood models. The impact of dose, dose-rate, and radiation quality has been studied with the goal of predicting radiological tissue injury. In this study, we determined the impact of aging on the gene expression profile of blood from mice exposed to radiation. Young (2 mo) and old (21 mo) male mice were irradiated with 4 Gy x-rays, total RNA was isolated from whole blood 24 h later, and subjected to whole genome microarray analysis. Pathway analysis of differentially expressed genes revealed young mice responded to x-ray exposure by significantly upregulating pathways involved in apoptosis and phagocytosis, a process that eliminates apoptotic cells and preserves tissue homeostasis. In contrast, the functional annotation of senescence was overrepresented among differentially expressed genes from irradiated old mice without enrichment of phagocytosis pathways. Pathways associated with hematologic malignancies were enriched in irradiated old mice compared with irradiated young mice. The fibroblast growth factor signaling pathway was underrepresented in older mice under basal conditions. Similarly, brain-related functions were underrepresented in unirradiated old mice. Thus, age-dependent gene expression differences should be considered when developing gene signatures for use in radiation biodosimetry.

## Introduction

The goal of radiation biodosimetry in a large-scale radiation emergency is to accurately estimate radiation dose, as a surrogate of radiological injury. A wide range of methods have been used to correlate cellular responses to radiation dose, ranging from cytogenetic measurements, such as dicentric and cytokinesis-block micronucleus assays, and γH2AX foci enumeration to high throughput proteomic, metabolomic, transcriptomic, and more recently, miRNA profiling^1,2^. We and others have reported gene expression-based analyses, with the development of genesets and pathways that correlate with dose, dose-rate, and radiation quality in both human and mouse blood^3–11^.

Aging is characterized by a progressive loss of cellular homeostasis, a decline in physiological function, and an increase in various pathologies, including cardiovascular disorders, neurodegenerative diseases, and cancer, which lead to increased morbidity and mortality^12–15^. Moreover, aging is associated with an overall decline in immune function that involves both the adaptive and the innate immune system^16–19^, which is attributed to the general deterioration of regenerative potential of aged hematopoietic stem cells. A fundamental aging mechanism that contributes to age-related dysfunction and chronic diseases is cellular senescence, which, in turn, promotes chronic inflammation thought to be the major cause of many age-related disorders, including atherosclerosis, autoimmunity, cancer, and dementias^20,21^. In aged individuals, immune system alterations, collectively called immunosenescence, result in a dampened adaptive and innate immune response, an increased pro-inflammatory response, enhanced susceptibility to infections and impaired efficacy of vaccination, as well as an increased production of autoantibodies^19,22^. Moreover, older individuals exhibit a low-level chronic inflammation called Inflammaging. Inflammaging, the association between aging and increased inflammation, has been attributed, at least in part, to the inability of a dysfunctional immune system to effectively clear pathogens and damaged host cells that lead to sustained inflammation^23,24^.

Aging is thought to be driven, at least in part, by the accumulation of stochastic damage in cells, attributed to the age-related functional loss in DNA damage response and repair^25,26^, leading to growth arrest and senescence^27^. Furthermore, nuclear DNA damage triggers a chronic inflammatory response^28–30^. As a result of unrepaired DNA double strand breaks, basal γH2AX foci increase with age in multiple human tissue types, as well as in senescent cells^31^, including fibroblasts and lymphocytes^32,33^. Furthermore, the spontaneous micronucleus yield in lymphocyte cultures from healthy donors aged 0–82 years correlates positively with increasing age, with an approximately fourfold increase in micronuclei in cultures from 80-year-old donors when compared to cultures from newborn donors^34^. Therefore, assessment of radiation dose based on γH2AX foci and micronuclei enumeration after exposure to radiation will be altered by the age of the affected individual. Furthermore, numerous studies have shown that age can modify basal gene expression patterns in both human and mouse tissues^35–38^. Extending these observations, we hypothesize that age will interfere with radiation biodosimetry that is based on transcriptomic changes in response to radiation.

In the current study, we evaluated the effect of aging on the response to radiation using a mouse model. We used a sub-lethal dose of total body irradiation and assessed global gene expression profiles in whole blood from young and old mice using Agilent Mouse Whole Genome microarrays. We used several class prediction methods to test whether age influenced the performance of a previously published gene expression signature^39^ to predict exposure to radiation. Furthermore, we applied bioinformatics analysis to identify differentially represented pathways, diseases, and functions. The results from this study will help define a radiation biodosimetry consensus gene signature that is valid across various age groups.

## Results

### Effect of radiation on blood cell counts of young and old mice

**Figure 1:**
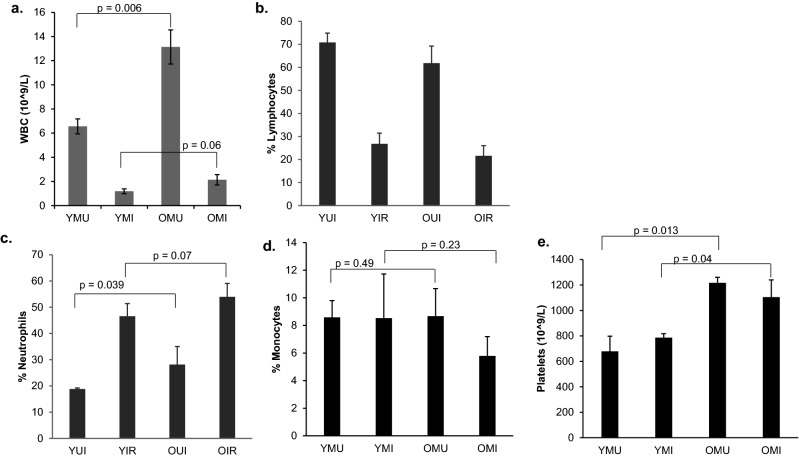
Total white blood cell count (WBC) and percentages of different blood cell types in young and old mice after 1 day of radiation exposure (4 Gy). (**a**) total WBC counts, (**b**) lymphocytes (%), (**c**) neutrophils (%), (**d**) monocytes (%), (**e**) platelets counts. Data represent the mean ± S.E.M. (n = 3). *P* values were calculated using the unpaired Student’s t-test. *YUI* young unirradiated, *YIR* young irradiated, *OUI* old unirradiated, *OIR* old irradiated.

We analyzed the differential expression of genes in whole blood from young (2 mo) and old (21 mo) male mice that had been exposed or not to 4 Gy x-ray irradiation. Overall, the number of white blood cells (WBC) in older mice was significantly higher than in younger mice (*p* = 0.006) (Fig. 1). However, the total number of WBC 24 h post-irradiation was similar in both age groups (*p* = 0.06). The relative percentage of lymphocytes in unirradiated cells from both young and old mice was 60–70% of the total WBC number, whereas after irradiation lymphocyte percentages fell to approximately 20% in both groups. In contrast, neutrophils, which are less sensitive to radiation than lymphocytes, became the most abundant species, accounting for 50–60% of the WBC after irradiation. While there was a statistically significant difference in the percentage of neutrophils from young and old mice before irradiation (*p* = 0.039), the difference was no longer significant after irradiation (*p* = 0.07). Moreover, percentages of monocytes, which differentiate to macrophages and dendritic cells, were similar before (*p* = 0.49) and after (*p* = 0.23) irradiation between young and old mice. In accordance with published reports^40^, platelet counts were found to be significantly elevated in older mice compared with young mice (*p* = 0.013) under basal conditions. However, unlike lymphocytes and neutrophils, exposure to radiation did not change platelet counts appreciably in either age group. This is consistent with published reports that platelets numbers diminish at a later time (~ 10 days) after irradiation^41^.

### Microarray analysis

**Figure 2:**
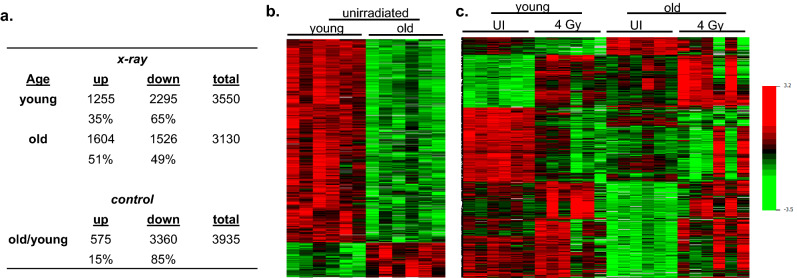
Differentially expressed genes. (**a**) Significantly differentially expressed genes in young and old mouse blood (*p* < 0.005) after 4 Gy x-rays (*x-ray*) relative to unirradiated mice and under basal conditions comparing old versus young mice (*control*). Percent of upregulated (*up*) and down-regulated (*down*) genes are shown. (**b**) Heatmap of gene expression changes between young and old mice under homeostatic conditions. (**c**) Heatmap depicting relative gene expression of age-related genes from (**b**) that respond significantly to irradiation. *Red* indicates high expression, *green* indicates low expression as indicated in the color key. Each row represents one gene and each column represents an individual mouse, ordered by exposure dose and age group as labeled in the figure. *UI* unirradiated.

Global gene expression was measured in the blood of young and old C57BL/6J male mice using Agilent’s whole mouse genome microarrays. Class comparison using BRB-ArrayTools^42^ identified a total of 3550 and 3130 differentially expressed genes (*p < 0*.005, false discovery rate (FDR) < 5%) in young irradiated versus young control mice and old irradiated versus old control mice, respectively (Fig. 2a; the complete list of differentially expressed genes can be found in Supplementary File 1). In agreement with our previous results^7^, 35% of the differentially expressed genes (DEGs) in the young mice were upregulated, whereas 65% were downregulated. In the old mice, 51% of the DEGs were upregulated and 49% were downregulated. A total of 5004 genes were responsive to ionizing radiation in at least one age group. Of these genes, 1,667 were common between young and old mice and the majority of these also displayed similar fold-changes between the two age groups, whereas 1883 genes (53%) in the “young” cohort and 1461 genes (47%) in the “old” cohort were unique to the respective age group. Furthermore, comparing the transcriptomic profile of old-control mice with young-control mice, we identified 3935 genes differentially expressed between the two age groups (Fig. 2b). The majority (85%) of these DEGs were downregulated in the old population compared with the young population. Out of the total number of age-related genes, 720 genes (18%) were also responsive to radiation (Fig. 2c).

### Impact of age on prediction of radiation exposure

Next, we used the class prediction tool in BRB-ArrayTools^42^ to develop a gene signature that would correctly detect radiation exposure. The greedy pairs method^43^ was used to identify the 12 top-performing pairs of genes for discrimination between irradiated and control samples. Each age group was used in turn as the training set for the gene selection process with the remaining samples serving as a test set. Despite the limited overlap between young and old gene sets, each training set was capable of distinguishing between irradiated and unirradiated samples with 100% accuracy in old and young mice, using seven independent classifier algorithms (Supplementary File 2). In a previous report^39^, we constructed a gene expression signature that combined samples from wildtype (WT) and two DNA repair mutant (*Atm*^*-/-*^ and *Prkdc*^*scid*^) mouse models that was able to detect exposure to an LD_50/30_ radiation dose with a 100% accuracy. We tested the ability of that signature to detect radiation exposure in young and old mice. Although only one gene, *Phlda3* (young) or *Cdkn1a* (old), was common between the earlier signature and either of the signatures reported here, we tested the so-called “mixed” (i.e., WT plus DNA repair deficient mutants) gene set on the dataset from this study and found that it was also able to detect radiation exposure in young mice with 100% efficiency (*p* < 0.001) (Table 1). However, the prediction of radiation exposure in older animals was less accurate, fluctuating between 58 and 67% (*p* < 0.001), depending on the classification method used. This data supports inclusion of individuals with a range of ages in gene selection to produce more robust signatures for radiation biodosimetry.

**Table 1:** Performance of seven classifier algorithms using greedy pairs gene selection with the “mixed” training set.

Test set	% Correct prediction						
	CCP	LDA	1-NN	3-NN	NC	SVM	BCCP
Young	100	100	100	100	100	100	100
Old	67	58	58	67	67	58	67

*CCP* compound covariate predictor, *LDA* linear discriminant analysis, *1-NN* 1-nearest neighbor, *3-NN* 3-nearest neighbors, *NC* nearest centroid, *SVM* support vector machines, *BCCP* Bayesian compound covariate predictor.

### Gene ontology analysis

We used Ingenuity Pathway Analysis (IPA) to identify the most significantly enriched canonical pathways and diseases or functions among differentially expressed genes considering a Benjamini-corrected *p* value of less than 0.05 to be significant. Moreover, the activation state of each process was determined by its *z*-score. A z-score of at least 2 indicated activation, whereas a z-score of -2 or less indicated inactivation/inhibition. Based on these criteria, we compared gene expression in irradiated young and old mice, as well as unirradiated young and old mice.

### Irradiated old versus irradiated young mice

Not surprisingly, young and old mice exposed to irradiation shared many canonical pathways (Supplementary File 3). These included the Integrin and IL-8 signaling pathways, which were both enriched in upregulated genes, or EIF2 and B cell activating factor signaling, which were both overrepresented among downregulated genes (Table 2). However, the two age groups presented some notable differences, as well. Thus, “Cytotoxic T Lymphocyte-mediated Apoptosis of Target Cells” was overrepresented in irradiated young mice (*p* = 2.29E−03; z = 2.65), whereas a number of T cell co-stimulatory signaling pathways, such as CD27 (*p* = 4.90E−02; z = − 2.33) and 4-1BB (*p* = 4.68E−02; z = − 2.65) were significantly underrepresented in irradiated old mice (Table 2), suggesting old mice have impaired T cell activation in response to radiation stress. Furthermore, apoptosis signaling (*p* = 4.17E−04; z = 2.236) was characteristic of irradiated young, but not irradiated old, mice. In addition, young mice responded to x-ray exposure by significantly upregulating genes involved in phagocytosis, a crucial process that eliminates apoptotic cells and preserve tissue homeostasis^44^. “Fcγ Receptor-mediated Phagocytosis in Macrophages and Monocytes” (*p* = 3.47E−02; z = 2.2) pathway was overrepresented among the upregulated differentially expressed genes from young irradiated mice (Table 2). Phagosome maturation (*p* = 1.32E−03) and phagosome formation (*p* = 3.09E−07) were significantly overrepresented specifically in irradiated young mice (Supplementary File 3). Phagocytosis-related pathways were not significantly over-represented among radiation responsive genes in old mice or in genes differentially expressed in unirradiated young versus old mice.

**Table 2:** Significant canonical pathways among genes differentially expressed in irradiated old and young male mice as identified by Ingenuity Pathway Analysis.

YOUNG mice			OLD mice		
Ingenuity canonical pathways	z-score	*p *value	Ingenuity canonical pathways	z-score	*p *value
**Upregulated**					
Integrin Signaling	3.539	3.02 E−06	Integrin Signaling	4.529	9.77 E−06
Actin Cytoskeleton Signaling	2.343	1.86 E−05	Relaxin Signaling	3.300	4.57 E−04
IL-8 Signaling	3.363	1.07 E−04	PPARα/RXRα Activation	2.353	5.89 E−04
Apoptosis Signaling	2.236	4.17 E−04	Cardiac Hypertrophy Signaling	3.000	1.86 E−03
P2Y Purigenic Receptor Signaling Pathway	2.502	5.25 E−04	eNOS Signaling	2.985	2.19 E−03
Rac Signaling	2.200	6.46 E−04	P2Y Purigenic Receptor Signaling Pathway	2.711	3.47 E−03
Regulation of Cellular Mechanics by Calpain Protease	2.333	1.51 E−03	Agrin Interactions at Neuromuscular Junction	2.138	5.01 E−03
Cytotoxic T Lymphocyt E−mediated Apoptosis of Target Cells	2.646	2.29 E−03	GP6 Signaling Pathway	2.858	7.08 E−03
Ephrin Receptor Signaling	2.524	2.69 E−03	Sperm Motility	2.294	7.08 E−03
RhoA Signaling	2.294	6.92 E−03	Colorectal Cancer Metastasis Signaling	2.263	1.26 E−02
Relaxin Signaling	2.524	1.15 E−02	Regulation of eIF4 and p70S6K Signaling	2.121	1.41 E−02
Cardiac β-adrenergic Signaling	2.000	1.62 E−02	Role of NFAT in Cardiac Hypertrophy	2.121	1.48 E−02
Fcγ Receptor-mediated Phagocytosis in Macrophages and Monocytes	2.183	3.47 E−02	Leptin Signaling in Obesity	2.236	3.55 E−02
Agrin Interactions at Neuromuscular Junction	2.309	4.79 E−02	Signaling by Rho Family GTPases	2.043	3.89 E−02
			IL-8 Signaling	3.272	4.27 E−02
			Ephrin Receptor Signaling	3.000	4.79 E−02
			AMPK Signaling	2.711	4.90 E−02
**Downregulated**					
Calcium-induced T Lymphocyte Apoptosis	− 2.711	2.51 E−08	EIF2 Signaling	− 3.138	5.13 E−04
iCOS-iCOSL Signaling in T Helper Cells	− 2.268	2.19 E−07	Role of CHK Proteins in Cell Cycle Checkpoint Control	− 2.333	6.61 E−04
EIF2 Signaling	− 3.430	2.95 E−07	B Cell Activating Factor Signaling	− 3.317	8.13 E−04
Role of CHK Proteins in Cell Cycle Checkpoint Control	− 2.111	4.90 E−04	April Mediated Signaling	− 2.714	1.86 E−03
PKCα¸ Signaling in T Lymphocytes	− 2.502	5.01 E−04	iCOS-iCOSL Signaling in T Helper Cells	− 2.236	2.24 E−03
B Cell Activating Factor Signaling	− 2.333	1.51 E−02	PKCα¸ Signaling in T Lymphocytes	− 2.449	1.66 E−02
			RhoGDI Signaling	− 2.132	2.00 E−02
			LPS/IL-1 Mediated Inhibition of RXR Function	− 2.714	2.82 E−02
			4-1BB Signaling in T Lymphocytes	− 2.449	4.68 E−02
			CD27 Signaling in Lymphocytes	− 2.333	4.90 E−02

Pathways displaying a z-score greater than 2 or lower than -2 and showing Benjamini-corrected *p* value < 0.05 were considered significant. Canonical pathways are ordered according to their *p* values.

“Disease and Functions” annotation (IPA) revealed that upregulated genes in irradiated mice were strongly associated with tumorigenesis (young, *p* = 2.90E−33; z = 4.425; old, *p* = 5.09E−24; z = 4.291) (Table 3; Supplementary File 4). However, a closer scrutiny of significant cancer-related functions revealed that genitourinary cancer-related biofunctions were enriched in irradiated young mice with the exception of prostate cancer disease that was over-represented in irradiated old mice (Supplementary Table S1). Furthermore, numerous hematologic-related proliferative diseases and neoplasms were predominantly enriched among upregulated genes in irradiated old mice compared with irradiated young mice (Supplementary Table S1). A number of known proto-oncogenes, including *Bcl3*^45^ (0.47-fold), *Bcl6*^46^ (0.56-fold), *Bcl9*^47^ (0.24-fold), and *Bcl11b*^48^ (0.22-fold) (Supplementary File S1), which are involved in hematological and epithelial malignancies, were downregulated in young mice exposed to x-rays, whereas, no such downregulation was recorded in the irradiated old mice. Cell death/apoptosis-related functions were markedly enriched in irradiated young mice, whereas they were almost completely absent in irradiated old mice (Supplementary Table S2). Notably, important pro-apoptotic genes such as *Bax* (2.35-fold), *Bak* (1.75-fold), and *Puma* (2.4-fold) were elevated only in irradiated young mice, but not irradiated old mice (Supplementary File 1). Elimination of lymphocytes is dependent upon the receptor Fas (CD95) and its cognate ligand Fasl (CD95L)^49^. *Fas* was overexpressed (1.71-fold) in young versus old mice at basal levels. When challenged with radiation, *Fasl* was upregulated (3.34-fold) in young mice, but not in old mice. Correspondingly, functions associated with quantity, proliferation, and viability of various immune cell subtypes were overrepresented among downregulated genes, mainly in the irradiated young animals, although several of them were significant in the irradiated old mice as well (Table 3; Supplementary Table S2). As a consequence, organismal death function was upregulated in irradiated young mice (*p* = 1.07E−34; z = 2.303), whereas morbidity and mortality (*p* = 2.79E−16; z = − 2.355) and organismal death (*p* = 1.12E−15; z = − 2.681) functions were significantly underrepresented in old mice exposed to radiation (Supplementary Table S2). Instead, irradiation of old mice led to enrichment of senescence function (*p* = 3.92E−05; z = 2.54). Furthermore, functions like repair of DNA appeared compromised in older mice exposed to radiation (*p* = 7.05E−8; z = − 3.52), whereas formation of nuclear foci (*p* = 3.42E−06; z = 2.20) was significantly elevated in irradiated old mice (Table 2; Supplementary File 4). However, despite cell death/apoptosis functions being underrepresented in differentially expressed genes from irradiated old mice, “quantity of hematopoietic progenitor dells” (*p* = 9.42E−08; z = − 2.294) and “proliferation of hematopoietic progenitor cells” (*p* = 1.72E−07; z = − 2.224) were significantly enriched among downregulated genes in irradiated old mice, but not irradiated young mice, implying the pool of hematopoietic stem (HSC) and progenitor cells may be more vulnerable to stress and/or unable to mobilize stem cells into differentiating into mature immune cells. Markedly, several signaling pathways known to provide protection to HSCs in response to stress or to mobilize HSCs to replenish damaged immune cells were enriched in irradiated young but not old mice. EGF signaling (*p* = 3.7E−03), IL-3 signaling (*p* = 5.5E−03), and GM-CSF signaling (7.59E−03) pathways were all overrepresented among upregulated genes in irradiated young animals, but not irradiated old ones. IL-7 (young, *p* = 6.2E−07; old, *p* = 1.4E−02) and IL-4 (young, *p* = 9.3E−04; old, *p* = 1.2E−02), signaling pathways that promote lymphocyte generation and survival after stress, were several orders of magnitude more significant in irradiated young versus irradiated old mice. None of these signaling pathways were differentially regulated in unchallenged, control young or old mice, although epidermal growth factor receptor (*Egfr*)*,* as well as the *Egfr*-related gene *Erbb2,* was downregulated (fivefold) in older mice compared with young mice (Supplementary File 1).

**Table 3:** Ten most significant (ordered by *p* value) diseases and functions enriched among upregulated or downregulated genes in irradiated old and young male mice as identified by Ingenuity Pathway Analysis.

YOUNG mice			OLD mice		
Diseases or functions annotation	z-score	*p* value	Diseases or functions annotation	z-score	*p* value
**Upregulated**					
Cancer	4.425	2.90 E−33	Cancer	4.291	5.09 E−24
Development of vasculature	4.348	3.43 E−18	Cell movement	3.546	1.04 E−18
Angiogenesis	4.348	8.06 E−18	Cell movement of blood cells	3.519	1.31 E−10
Endocytosis	5.272	3.03 E−17	Adhesion of blood cells	3.564	5.08 E−07
Phagocytosis	6.210	1.77 E−15	Cell spreading	3.653	6.79 E−07
Phagocytosis of cells	5.691	6.41 E−15	Cell movement of leukocytes	3.542	2.72 E−06
Engulfment of cells	5.425	9.99 E−15	Cell death of osteosarcoma cells	4.596	7.82 E−06
Internalization of cells	5.053	1.28 E−13	Homing of cells	3.810	8.59 E−06
Phagocytosis of blood cells	4.331	2.10 E−11	Chemotaxis	3.778	2.61 E−05
Endocytosis by eukaryotic cells	4.882	6.37 E−11	Adhesion of immune cells	3.510	4.78 E−05
**Downregulated**					
Quantity of lymphatic system cells	− 3.674	1.07 E−52	Quantity of IgG	− 3.802	9.33 E−12
Quantity of mononuclear leukocytes	− 3.402	1.93 E−49	Quantity of B lymphocytes	− 3.301	2.34 E−11
Quantity of lymphoid cells	− 3.418	4.70 E−49	Repair of DNA	− 3.519	7.05 E−08
Quantity of lymphocytes	− 3.522	8.02 E−49	Quantity of IgG1	− 3.473	7.74 E−08
Quantity of immunoglobulin	− 3.153	1.41 E−28	Checkpoint control	− 3.229	1.68 E−07
Quantity of B lymphocytes	− 4.263	3.89 E−27	Hemorrhagic disease	− 3.450	2.07 E−07
Quantity of lymphoid tissue	− 3.855	2.10 E−25	Development of B lymphocytes	− 2.995	2.09 E−06
Quantity of IgG	− 4.221	3.93 E−22	Stabilization of chromosomes	− 3.246	2.68 E−06
Quantity of IgG1	− 3.318	1.39 E−14	Excision repair	− 3.246	1.27 E−05
Antibody response	− 3.123	3.92 E−14	Bleeding	− 3.847	2.50 E−05

Benjamini-corrected p value < 0.05 and |z|≥ 2 was considered significant.

**Figure 3:**
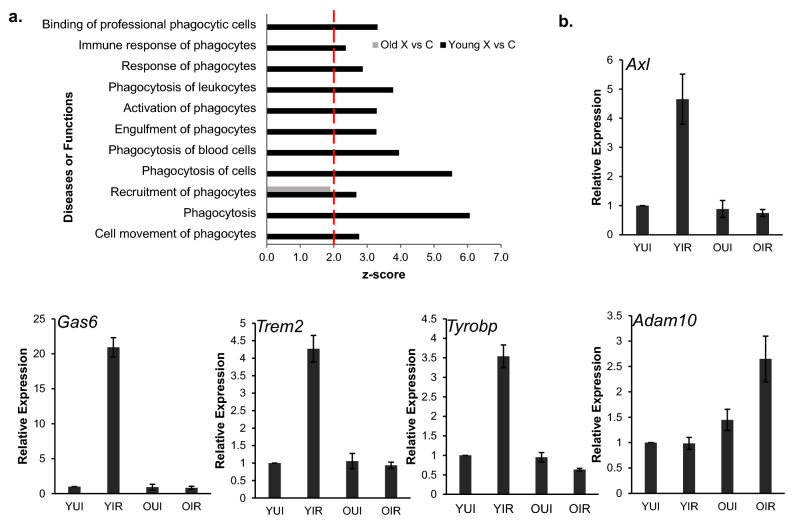
Phagocytosis-related biological functions are overrepresented in irradiated young male mice. (**a**) Significantly enriched phagocytosis-related functions in young-irradiated versus old-irradiated mice. X: x-ray; C: control (unirradiated). Dotted line marks z-score = 2.0. (**b**) Gene expression levels of 5 phagocytosis-related genes (*Axl*, *Gas6*, *Trem2*, *Tyrobp,* and *Adam10*) analyzed by RT-qPCR and normalized to *Actb* expression. Data represent the mean ± S.E.M. (n = 3). *YUI* young unirradiated, *YIR* young irradiated, *OUI* old unirradiated, *OIR* old irradiated.

Lastly, numerous phagocytosis-related functions were significantly overrepresented among upregulated genes in irradiated young mice (Table 3 and Fig. 3a), whereas they were absent from irradiated old mice. Genes with known roles in phagocytosis, including *Axl* (3.4-fold) and its ligand *Gas6* (growth arrest specific 6; 17.2-fold), *Trem2* (triggering receptor expressed on myeloid cells 2; twofold) and *Tyrobp/Dap12* (TYRO protein tyrosine kinase-binding protein/ DNAX-activating protein of 12 kDa; 2.2-fold), were upregulated exclusively in irradiated young mice. In contrast, the phagocytosis inhibitor *Adam10* (ADAM metallopeptidase domain 10; 2.2-fold) was upregulated in irradiated old mice (Supplementary File 1). To validate the microarray findings, we analyzed expression of these genes by quantitative-PCR. We determined that *Axl* (4.6-fold), *Gas6* (20-fold), *Trem2* (4.2-fold), and *Tyrobp* (3.5-fold) were upregulated in irradiated young, but not old mice, whereas *Adam10* (2.6-fold), was expressed at higher levels in irradiated old mice (Fig. 3b). In contrast, none of these genes was differentially expressed under basal conditions.

### Unirradiated old versus unirradiated young mice

IPA analysis demonstrated that the majority of the canonical pathways were underrepresented in old compared with young mice (Table 4; Supplementary File 3). The most significant pathway was Fibroblast growth factor (Fgf) signaling (*p* = 3.70E−04; z = − 2.8). Numerous *Fgf* coding genes were downregulated in older mice (Supplementary Table S3). However, the Fgf pathway was not involved in the response to irradiation, except for FGF receptor 1 (*Fgfr1*), which was significantly increased in irradiated young mice (11.7-fold). In addition to Fgf signaling, sperm motility (*p* = 2.95E−03; z = − 2.985) and GnRH (gonadotropin-releasing hormone-like protein) signaling (*p* = 3.72E−02; z = − 2.524), involved in spermatogenesis^50^, were significantly underrepresented in old mice. A majority of the X-linked reproductive homeobox (*Rhox*) and *Hox* gene clusters that play a role in male fertility^51^ was downregulated in old mice compared with young mice as visualized in a heatmap (Supplementary Fig. S1). Lastly, several nervous system pathways were underrepresented in the differentially expressed geneset from old mice compared with young mice, including the neuroprotective role of THOP1 (Thimet Oligopeptidase 1) in Alzheimer’s disease (*p* = 1.15E−02; z = − 3.153), neurotrophin/TRK (neurotrophic receptor tyrosine kinase 1) signaling (*p* = 1.26–02; z = − 2.887), and the Wnt/Ca^+^ signaling (*p* = 4.79E−03; z = − 2.673) pathways that contribute to neuron protection and regeneration.

**Table 4:** Canonical pathways significantly over-represented among downregulated genes in control old versus young male mice under basal (no irradiation) conditions as assessed by Ingenuity Pathway Analysis.

Ingenuity canonical pathways	z-score	*p* value
FGF signaling	− 2.837	3.72 E−04
Sperm motility	− 2.985	2.95 E−03
cAMP-mediated signaling	− 2.197	4.17 E−03
Wnt/Ca + pathway	− 2.673	4.79 E−03
GPCR-mediated nutrient sensing in enteroendocrine cells	− 2.837	5.62 E−03
Role of NFAT in cardiac hypertrophy	− 3.087	7.24 E−03
Synaptic long term depression	− 3.900	7.59 E−03
Neuropathic pain signaling in dorsal horn neurons	− 3.273	7.76 E−03
Neuroprotective role of THOP1 in Alzheimer's disease	− 3.153	1.15 E−02
Neurotrophin/TRK signaling	− 2.887	1.26 E−02
CREB signaling in neurons	− 3.545	1.29 E−02
Bladder Cancer Signaling	− 3.162	1.91 E−02
Actin Cytoskeleton Signaling	− 2.558	2.00 E−02
Basal Cell Carcinoma Signaling	− 2.530	3.47 E−02
GNRH Signaling	− 2.524	3.72 E−02
Endothelin-1 Signaling	− 2.268	3.89 E−02
ERK5 Signaling	− 2.714	3.89 E−02
Calcium Signaling	− 4.583	3.89E−02

Benjamini-corrected *p* value < 0.05 and |z|≥ 2 was considered significant.

**Figure 4:**
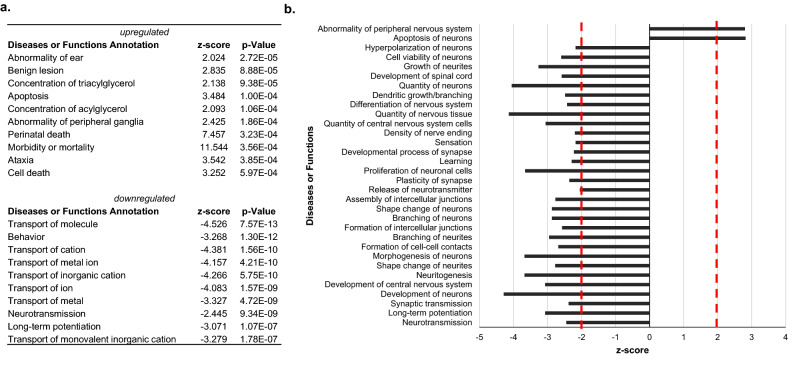
Diseases and functions significantly overrepresented in young versus old mice. (**a**) Top ten diseases and functions significantly enriched among upregulated and downregulated genes in control old versus young male mice. (**b**) Brain-related functions differentially overrepresented in young versus old animals. |z|> 2 is considered significant. Dotted red lines mark z-score = 2.0 or – 2.0.

Diseases and functions annotation revealed that, under basal conditions, cell death/apoptosis/morbidity, including neuron (*p* = 1.01E−03; z = 2.821) and embryonic cell (*p* = 1.43E−03; z = 2.329) death, as well as morbidity or mortality (*p* = 3.56E−04; z = 11.544) were significantly elevated in old unirradiated animals (Fig. 4a; Supplementary File 4). In contrast, molecular transport and brain development functions were under-represented in old mice compared with young animals (Fig. 4a). A systematic investigation of nervous system physiology-related functions revealed a great number of functions that were upregulated in young mice, whereas functions related to neuronal dysfunction and/or cell death (apoptosis) were markedly underrepresented in the young mice compared with old mice (Fig. 4b and Supplementary Table S4 for z-score values). Finally, unlike the response to stress, cancer differences were not apparent between young and old mice, although benign lesions/neoplasm-related diseases were more significant in old than young mice (Supplementary File 4).

## Discussion

An important goal of radiation biodosimetry is to accurately predict radiation dose exposure as a surrogate of severity of tissue injury. Previously, we described the gene expression profile in peripheral blood from mice exposed to x-ray radiation^7^. These results suggested that differences in gene expression could potentially be exploited to estimate radiation dose. However, several biological variables, including age, sex, and infection, could potentially modify the transcriptomic profile of an individual exposed to irradiation. In the present study, we have examined the impact of age on gene expression changes in male mice exposed to a single whole-body dose of x-rays. We have identified differentially regulated genes in response to x-ray radiation exposure and under homeostatic conditions and have categorized these genes into canonical pathways and diseases or functions.

A major finding of this study was the upregulation of phagocytosis in young mice exposed to radiation, but not in irradiated old mice. However, under basal conditions, neither young nor old mice demonstrated significant enrichment in phagocytosis-related functions. The correlation between phagocytosis with age under homeostatic conditions remains contentious with some studies showing that murine macrophage phagocytosis is not altered with age^52,53^, while others describe an impairment^54–56^, or even enhanced phagocytic activity^57–60^ with age. These differences could be attributed to different animal models and tissue sites.

Phagocytosis is crucial in maintaining tissue homeostasis and innate immune balance and it is tasked with the clearance of apoptotic cells, a process called efferocytosis, as well as invading pathogens such as bacteria and microbes^61^. Although epithelial and endothelial cells, as well as fibroblasts, are able to phagocytose apoptotic cell debris, phagocytosis is predominantly carried out by the so-called professional phagocytes, including macrophages, dendritic cells, and neutrophils. Dysfunctional phagocytosis leads to accumulation of unphagocytosed debris with subsequent accumulation of secondary necrotic debris that promotes chronic inflammation and autoimmune diseases^62^, such as systemic lupus erythematosus^63,64^, and exacerbates tissue damage. Therefore, phagocytosis has been considered a key requirement for inflammatory resolution and for the preservation of immune tolerance^65^. Moreover, a diminished phagocytic capacity of dendritic cells impairs antigen presentation to T cells and activation of the adaptive immune system^66^. In brain, microglial function has been linked to maintenance of homeostasis through phagocytic clearance of cellular debris and protein aggregates, whereas they display functional deterioration during aging and promote neurodegenerative diseases^67^, including Alzheimer’s and Parkinson’s diseases^68^. Furthermore, retinal pigment epithelial cell (the resident phagocytes in the eye) dysfunction has been implicated in the pathogenesis of age-related macular degeneration and blindness^69^.

Phagocytosis is a receptor-mediated process^70^. Receptors involved in the recognition and uptake of apoptotic bodies belong to the Tyro3-Axl-Mer (TAM) tyrosine kinase proteins. TAM receptors are functionally involved in two discrete phenomena: the phagocytosis of apoptotic corpses and the regulation of the innate immune response^70^. TAM receptors rely on their cognate activating ligands, Gas6, which activates predominantly Axl, and protein S (Pros1), with specificity for Mer and Tyro3, for their phagocytic activity^71^. Gas6 and Pros1 drive phagocytosis only when they are simultaneously bound to a TAM receptor and phosphatidylserine, which serves as an “eat-me” signal in phagocytosis^71^. Ablation of Axl and Mer in TAM knockout mice leads to increased inflammation^72,73^. In the past, Axl and Mer kinases were thought to act redundantly in phagocytosis; however, accumulating evidence suggests that Mer preferentially mediates the phagocytosis of apoptotic cells under homeostasis, whereas Axl specifically controls the process under stress, such as infection^74,75^. Our microarray data show that *Axl* but not *Mer* is upregulated in irradiated young mice supporting distinct roles of these two kinases in response to a different kind of stress, namely radiation. In brain, TAM receptors involved in phagocytosis of apoptotic debris can be engaged on Trem2-expressing microglia, the resident macrophages of the central nervous system^76^. Trem2-ligand interaction recruits Tyrobp/Dap12 and mediate signal transduction^68^. When phagocytosis is completed, Trem2 is released from the surface of cells through the consecutive action of Adam10 and γ-secretase that cleave Trem2, release Tyrobp/Dap12 and terminate Trem2 signaling^77^. Trem2 deletion or impairment in mice and in in vitro cell cultures reduces phagocytosis of apoptotic cells, cellular debris, lipoproteins, Aβ, and bacteria^78,79^. Importantly, human TREM2 shedding by cleavage is accelerated in Alzheimer’s disease^80,81^. Furthermore, individuals homozygous for rare inactivating mutations in either TREM2 or TYROBP develop a lethal form of progressive, early-onset dementia known as Nasu-Hakola disease^82,83^. Here, we show that *Trem2* and *Tyrobp* were significantly upregulated in irradiated young mice, whereas *Adam10* was elevated in irradiated old mice, emphasizing a potentially vital role of phagocytosis in brain after radiation exposure.

Apoptosis plays multiple critical roles in the immune system, such as the negative selection of thymocytes and lymphocytes, as a defense mechanism against autoimmunity, and in the maintenance of proliferative homeostasis^49^. Furthermore, proliferating hematopoietic system cells predominantly undergo apoptosis in response to irradiation^84^, which are removed in time by professional and non-professional phagocytes. Our analysis showed that the gene expression profile in peripheral blood cells from young mice exposed to irradiation correlates with an upregulation of apoptosis-related functions, whereas functions associated with quantity of many immune cell subtypes are markedly underrepresented. In contrast, cell death or apoptosis was not an important function of irradiated old mice. Instead, a senescence-related function appeared to predominate in these cells. Likewise, decreased DNA repair and persistent nuclear foci formation after irradiation are detected in older individuals^31–33^. Based on these results, we speculate that aging is associated with increased senescence, but decreased apoptosis and enhanced survival of functionally defective cells after DNA damage, which may lead to induction of inflammation^85^. In contrast, radiation induces apoptosis in blood cells from young mice that subsequently are cleared by phagocytosis leading to inflammatory resolution and restoration of immune system function. However, functional studies will be necessary to confirm this hypothesis.

Aging is characterized by dysregulation of hematopoietic stem cells (HSCs) and inefficient hematopoiesis^86^. Specifically, it has been demonstrated that the functional capabilities of HSCs and progenitor cells are grossly impaired and cannot respond efficiently to stress that disturbs homeostatic immune cell balance, such as infection, inflammation, or bone marrow transplantation^86^. Our transcriptomic analysis revealed that several stem/progenitor cell-related pathways were underrepresented in irradiated old mice, but not irradiated young mice. Furthermore, a number of growth factor and cytokine signaling pathways known to protect HSCs and progenitor cells from radiation is shown here to be significantly overrepresented in irradiated young mice. For example, it has been reported that EGF treatment protects bone marrow HSCs in response to stress^87^. We demonstrate that EGF signaling pathway is significantly enriched in irradiated young mice, but not irradiated old mice. Moreover, *Egfr* gene transcript level was higher in young mice compared with old ones. We also discovered that IL-3 and GM-CSF signaling pathways are enriched in young mice exposed to DNA damage, but not irradiated old mice. Both pathways can influence the survival and differentiation of HSCs toward myeloid lineages by activating transcription factors, while they stimulate emergency granulopoiesis during infection and injury^88,89^. Likewise, signaling pathways known to promote lymphocyte generation and CD4/CD8 ratio normalization after stress, such as IL-7 and IL-4^90,91^, were several orders of magnitude more significant in young irradiated animals compared with older ones. In contrast, none of the ‘stem cell”-related or cytokine signaling pathways were differentially regulated in unirradiated young or old mice.

Under homeostatic conditions, Fibroblast growth factor (Fgf) was the most significant pathway underrepresented in old mice as opposed to young mice. Fgf signaling plays an essential role in almost every cell fate decision, patterning event and coordinated cell movement in the early embryo. In the adult, FGFs are homeostatic factors functioning in tissue repair, wound healing, control of the nervous system and tumor angiogenesis^92^. Aberrant activity of the pathway is associated with developmental defects that disrupt organogenesis, impair the response to injury, and result in metabolic disorders, and cancer^93^. Several Fgf’s have been tested therapeutically to mitigate various pathologies, including human FGF18 for the treatment of osteoarthritis^94^ and recombinant human FGF2 gene therapy for cardiovascular disease^95^. In addition, recombinant human FGF7 (and FGF20) has been used to treat mucositis in response to radiation therapy or as a medical countermeasure against radiation exposure^96–100^. All these genes were found downregulated in old compared with young mice.

Cancer-related diseases were significant in both irradiated young and old animals. However, genitourinary tumor functions were associated with irradiated young mice, although prostate cancer functions were overrepresented in irradiated old mice. Similarly, various hematologic proliferative diseases and malignancies were more prevalent in irradiated old mice. However, under basal conditions, functions related with neurogenesis were significantly underrepresented in older mice compared with younger mice. In contrast to old mice, young mice demonstrated an enrichment in functions associated with diminished neuronal death and increased neuron activity and development, including release of neurotransmitters. In contrast, functions related to neurogenesis or brain function were not significantly over-represented among responding genes in old or young mice 24 h after irradiation.

In conclusion, we show that aging promotes large-scale rewiring of transcriptional networks in male mice exposed to radiation. Whereas we describe a number of pathways that are differentially overrepresented between young and old mice exposed to irradiation and could potentially serve to develop medical countermeasures, the most prominent functions and diseases that distinguish exposure to radiation between old and young mice are “induction of cell death/apoptosis” and “phagocytosis” that are significantly overrepresented in irradiated young mice, but not irradiated old animals. Interestingly, the link between increased apoptosis and diminished late toxicity in cancer patients who have undergone radiotherapy has been known for some time^101^. Furthermore, increased resistance to apoptosis has been associated with the aging process^85^. In the current study, in addition to apoptosis, we highlight the significance of phagocytosis in the response of young animals to radiation exposure. We postulate that irradiation causes an increase not only of apoptosis, but also of phagocytosis that helps clear apoptotic cells from the organism and thus reduce acute and persistent inflammation in response to damage. In contrast, functions related to “senescence”, “impaired DNA repair”, and “diminished quantity and proliferation of hematopoietic progenitor cells” are significantly overrepresented in irradiated old mice. Therefore, our long –term goal is to use apoptosis/phagocytosis as a molecular signature for the development of late toxicity. However, functional phagocytosis assays need be performed to confirm these findings, including performing longitudinal studies for phagocytosis activation, as it may be the timing of activation, rather than an absolute non-responsiveness, that occurs in the aged mice. Other potential variables such as sex and infectious status may also interact with age to alter transcriptional response, and these areas should also be examined. Sex may contribute to distinct gene expression responses to radiation^4^. In addition, age-related differences in the composition and activation of immune cells in females and males may also influence gene expression differentially^102^. Currently, we focus on elucidating the impact of age on gene expression in female mice exposed to radiation. Encouragingly, preliminary experiments confirm the observation that phagocytosis-related functions are overrepresented only in irradiated female young mice, but not irradiated old ones. Finally, class prediction analysis demonstrated that aging can be a confounding factor in accurate detection of radiation exposure. Therefore, future efforts are necessary to refine our gene expression signature taking age into account.

## Methods

### Animals and irradiation

A total of 24 male C57BL/6 mice were purchased from Jackson Labs and acclimated for 2 weeks before irradiation. We used 12 young (2 months) and 12 old (21 months) male mice. Each treatment group consisted of 6 mice and the treatment was as follows: (1) Control (unirradiated)-young male mice, (2) Control (unirradiated)-old male mice, (3) 4 Gy x-ray-young male mice, (4) 4 Gy x-ray-old male mice. Mice were fed the standard chow, without caloric restriction. All animal experiments were conducted in accordance with applicable federal and state guidelines based on approved by the Columbia University Institutional Animal Care and Use Committee (approval number AC-AAAT6450). Mice were restrained in a pie holder without anesthesia and they were either sham-irradiated or exposed to 4 Gy x-rays of total body irradiation from an X-RAD 320 Biological Irradiator (operating at 320 kV, 12.5 mA with a 2 mm Al filter [HVL ~ 1.0 mm Cu]) at a dose rate of 1 Gy/min, using the Radcal Accu-dose dosimeter with an ion chamber digitizer to monitor actual dose rate.

### Blood collection and RNA isolation

Blood was collected 1 day post-irradiation by cardiac puncture at the time of euthanasia (by CO_2_ asphyxiation). Each sample (~ 0.4 ml blood) was added to a 15 ml centrifuge tube that contained 1.6 ml of PAXgene Blood RNA stabilization and lysis solution (PreAnalytix GmBH) and mixed thoroughly, while a small amount of blood was added to sodium EDTA anti-coagulant containing tubes for blood count using a Genesis hematology system (Oxford Science). After collection, blood was incubated at 4 °C for 24 h. RNA was purified following the PAXgene RNA kit recommendations with on-column DNase I treatment. As high amounts of globin transcript have been shown to interfere with gene expression signatures derived from blood, globin RNA was reduced using the Ambion GLOBINclear-mouse/rat kit (Thermofisher). RNA yields were quantified using the NanoDrop ND1000 spectrophotometer (Thermofisher) and RNA quality was checked by the 2100 Bioanalyzer (Agilent). High quality RNA with an RNA integrity number of at least 7.0 was used for microarray hybridization.

### Microarray hybridization

Cyanine-3 labeled cRNA was prepared using the One-Color Low input Quick Amp Labeling kit (Agilent). Dye incorporation and cRNA yield was measured with a NanoDrop ND1000 spectrophotometer (Thermofisher). Labeled cRNA was fragmented and hybridized to Agilent Mouse Gene Expression 4 × 44 K v2 Microarray Kit (G4846A). Slides were scanned with the Agilent DNA microarray scanner (G2505B) and the images were analyzed with Feature Extraction software (Agilent) using default parameters for background correction and flagging non-uniform features.

### Data analysis

Background-corrected hybridization intensities were imported into BRB-ArrayTools, version 4.5.1, log_2_-transformed and median normalized. Non-uniform outliers or features not significantly above background intensity in 25% or more of the hybridizations were filtered out. In addition, a minimum 1.5-fold change in at least 20% of the hybridizations was set as a requirement. Furthermore, probes were averaged to one probe per gene and duplicate features were reduced by selecting the one with maximum signal intensity. Class comparison was conducted in BRB-ArrayTools to identify genes differentially expressed between radiation exposed samples and matched unirradiated controls using a random variance t-test^43^. Young versus old controls were also compared with each other. Genes with *p* values less than 0.005 were considered statistically significant. The false discovery rate (FDR) was estimated for each gene by the method of Benjamini and Hochberg^103^, to control for false positives. The cutoff in this analysis was set at an FDR of less than 0.05. Hierarchical clustering of microarray gene expression data was performed with the Dynamic Heatmap Viewer of the BRB-ArrayTools software using a one minus correlation metric and average linkage. Genes differentially expressed following exposure to 4 Gy x-ray at day 1 were used to construct the heatmap. Venn diagrams were used to identify unique and overlapping differentially expressed genes from irradiated old and young mice.

### Class prediction

The class prediction tool in BRB-ArrayTools was used to select genes and build predictors of radiation exposure status. The greedy pairs method^43^ was used to select the 12 pairs of genes that best discriminated between control and irradiated samples in each training set (young or old). After feature selection, seven classification methods (compound covariate predictor, linear discriminant analysis, 1- and 3-nearest neighbors, nearest centroid, support vector machines, and Bayesian compound covariate predictor) were used with the selected feature sets to predict the irradiation status of the remaining samples (old or young). The percentage correct classification was calculated for each approach. The same approach was repeated using the “mixed” (WT and repair mutants)^39^ gene signature as the training set and irradiated young and old data as the test set.

### Gene ontology analysis

Lists of genes that were either significantly overexpressed or underexpressed compared with controls were analyzed using the Ingenuity Pathway Analysis (IPA) core pathway (Qiagen Ingenuity Systems) to identify canonical pathways, diseases and functions. Benjamini corrected p values of < 0.05 were considered significant.

### Quantitative RT-PCR

A subset of the expression changes was validated by quantitative real-time PCR (RT-qPCR). cDNA was prepared from total globin-cleared RNA using the High-Capacity cDNA Archive kit (Thermofisher). RT-qPCR was performed using pre-designed validated Taqman assays (Thermofisher) for *Axl* (Mm00437221_m1), *Gas6* (Mm00490378_m1), *Trem2* (Mm04209422_m1), *Adam10* (Mm00545742_m1), *Tyrobp* (Mm00449152_m1). The gene expression validation experiments were conducted with 20 ng cDNA using Universal PCR Master Mix (Thermofisher) in a QuantStudio 7 Flex Real Time PCR system (Thermofisher). Relative fold-induction was calculated by the 2^−ΔΔCT^ method^104^, using QuantStudio Design & Analysis Software (Thermofisher). Data was normalized to β-actin gene (*Actb*; Mm00607939_s1) expression levels.

## Supplementary Information

Supplementary Figures and Tables.

Supplementary File 1.

Supplementary File 2.

Supplementary File 3.

Supplementary File 4.

## Data Availability

The microarray data generated in this study have been deposited in the National Center for Biotechnology Information Gene Expression Omnibus database with accession number GSE132559 (http://www.ncbi.nlm.nih.gov/geo/query/acc.cgi?acc=GSE132559).
