# Supplementary material for: Impact of aging on gene expression response to x-ray irradiation using mouse blood
Source: Sci Rep. 2021 May 13;11:10177. doi: 10.1038/s41598-021-89682-7 (PMC8119453; doi:10.1038/s41598-021-89682-7)
Supplement: Supplementary file 1 — Supplementary Figures and Tables. [file 41598_2021_89682_MOESM1_ESM.pptx]

## Slide 1
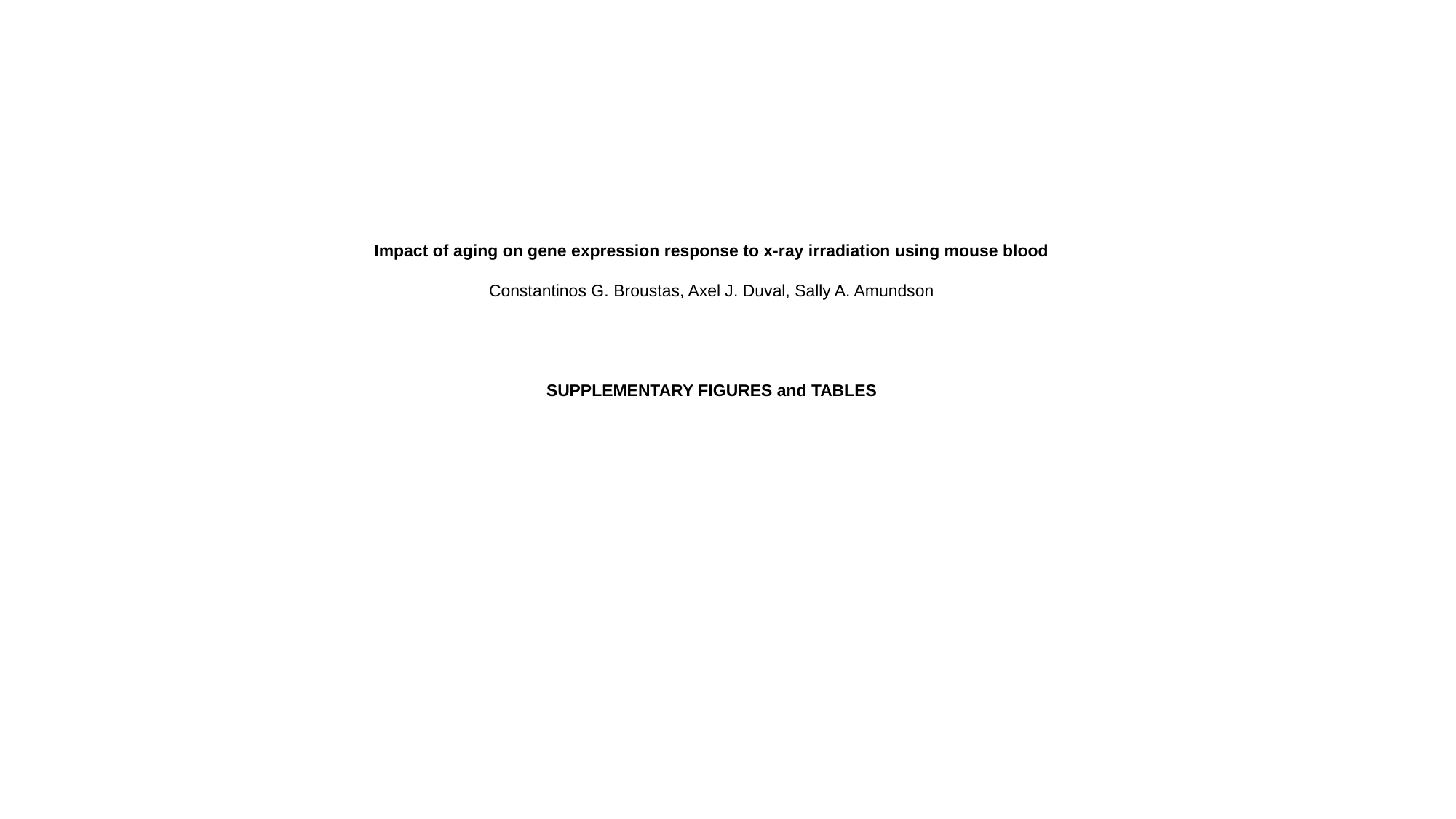

Impact of aging on gene expression response to x-ray irradiation using mouse blood
Constantinos G. Broustas, Axel J. Duval, Sally A. Amundson
SUPPLEMENTARY FIGURES and TABLES

## Slide 2
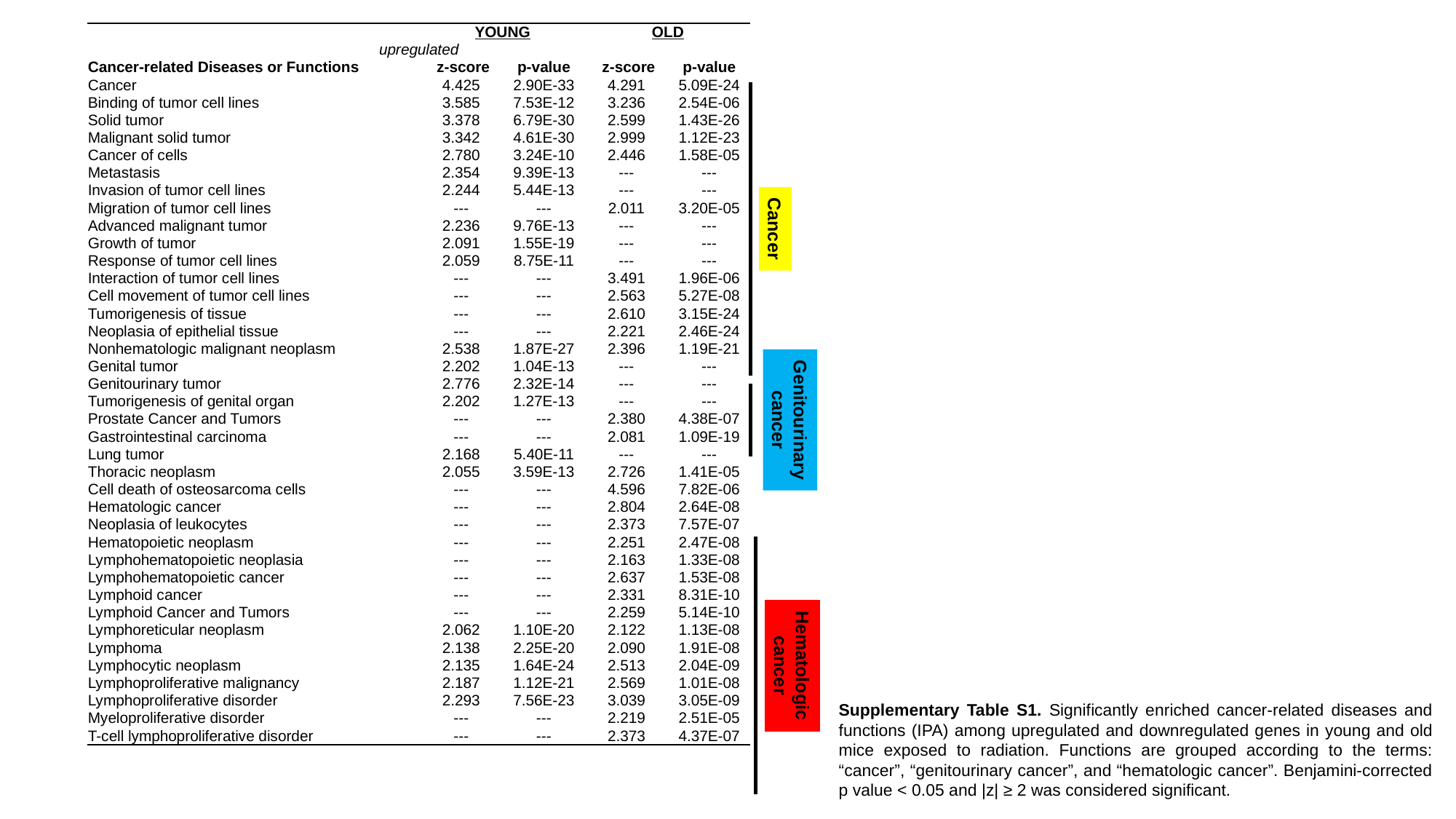

| | YOUNG | | OLD | |
| --- | --- | --- | --- | --- |
| upregulated | | | | |
| Cancer-related Diseases or Functions | z-score | p-value | z-score | p-value |
| Cancer | 4.425 | 2.90E-33 | 4.291 | 5.09E-24 |
| Binding of tumor cell lines | 3.585 | 7.53E-12 | 3.236 | 2.54E-06 |
| Solid tumor | 3.378 | 6.79E-30 | 2.599 | 1.43E-26 |
| Malignant solid tumor | 3.342 | 4.61E-30 | 2.999 | 1.12E-23 |
| Cancer of cells | 2.780 | 3.24E-10 | 2.446 | 1.58E-05 |
| Metastasis | 2.354 | 9.39E-13 | --- | --- |
| Invasion of tumor cell lines | 2.244 | 5.44E-13 | --- | --- |
| Migration of tumor cell lines | --- | --- | 2.011 | 3.20E-05 |
| Advanced malignant tumor | 2.236 | 9.76E-13 | --- | --- |
| Growth of tumor | 2.091 | 1.55E-19 | --- | --- |
| Response of tumor cell lines | 2.059 | 8.75E-11 | --- | --- |
| Interaction of tumor cell lines | --- | --- | 3.491 | 1.96E-06 |
| Cell movement of tumor cell lines | --- | --- | 2.563 | 5.27E-08 |
| Tumorigenesis of tissue | --- | --- | 2.610 | 3.15E-24 |
| Neoplasia of epithelial tissue | --- | --- | 2.221 | 2.46E-24 |
| Nonhematologic malignant neoplasm | 2.538 | 1.87E-27 | 2.396 | 1.19E-21 |
| Genital tumor | 2.202 | 1.04E-13 | --- | --- |
| Genitourinary tumor | 2.776 | 2.32E-14 | --- | --- |
| Tumorigenesis of genital organ | 2.202 | 1.27E-13 | --- | --- |
| Prostate Cancer and Tumors | --- | --- | 2.380 | 4.38E-07 |
| Gastrointestinal carcinoma | --- | --- | 2.081 | 1.09E-19 |
| Lung tumor | 2.168 | 5.40E-11 | --- | --- |
| Thoracic neoplasm | 2.055 | 3.59E-13 | 2.726 | 1.41E-05 |
| Cell death of osteosarcoma cells | --- | --- | 4.596 | 7.82E-06 |
| Hematologic cancer | --- | --- | 2.804 | 2.64E-08 |
| Neoplasia of leukocytes | --- | --- | 2.373 | 7.57E-07 |
| Hematopoietic neoplasm | --- | --- | 2.251 | 2.47E-08 |
| Lymphohematopoietic neoplasia | --- | --- | 2.163 | 1.33E-08 |
| Lymphohematopoietic cancer | --- | --- | 2.637 | 1.53E-08 |
| Lymphoid cancer | --- | --- | 2.331 | 8.31E-10 |
| Lymphoid Cancer and Tumors | --- | --- | 2.259 | 5.14E-10 |
| Lymphoreticular neoplasm | 2.062 | 1.10E-20 | 2.122 | 1.13E-08 |
| Lymphoma | 2.138 | 2.25E-20 | 2.090 | 1.91E-08 |
| Lymphocytic neoplasm | 2.135 | 1.64E-24 | 2.513 | 2.04E-09 |
| Lymphoproliferative malignancy | 2.187 | 1.12E-21 | 2.569 | 1.01E-08 |
| Lymphoproliferative disorder | 2.293 | 7.56E-23 | 3.039 | 3.05E-09 |
| Myeloproliferative disorder | --- | --- | 2.219 | 2.51E-05 |
| T-cell lymphoproliferative disorder | --- | --- | 2.373 | 4.37E-07 |
Cancer
Genitourinary
cancer
Hematologic
cancer
Supplementary Table S1. Significantly enriched cancer-related diseases and functions (IPA) among upregulated and downregulated genes in young and old mice exposed to radiation. Functions are grouped according to the terms: “cancer”, “genitourinary cancer”, and “hematologic cancer”. Benjamini-corrected p value < 0.05 and |z| ≥ 2 was considered significant.

## Slide 3
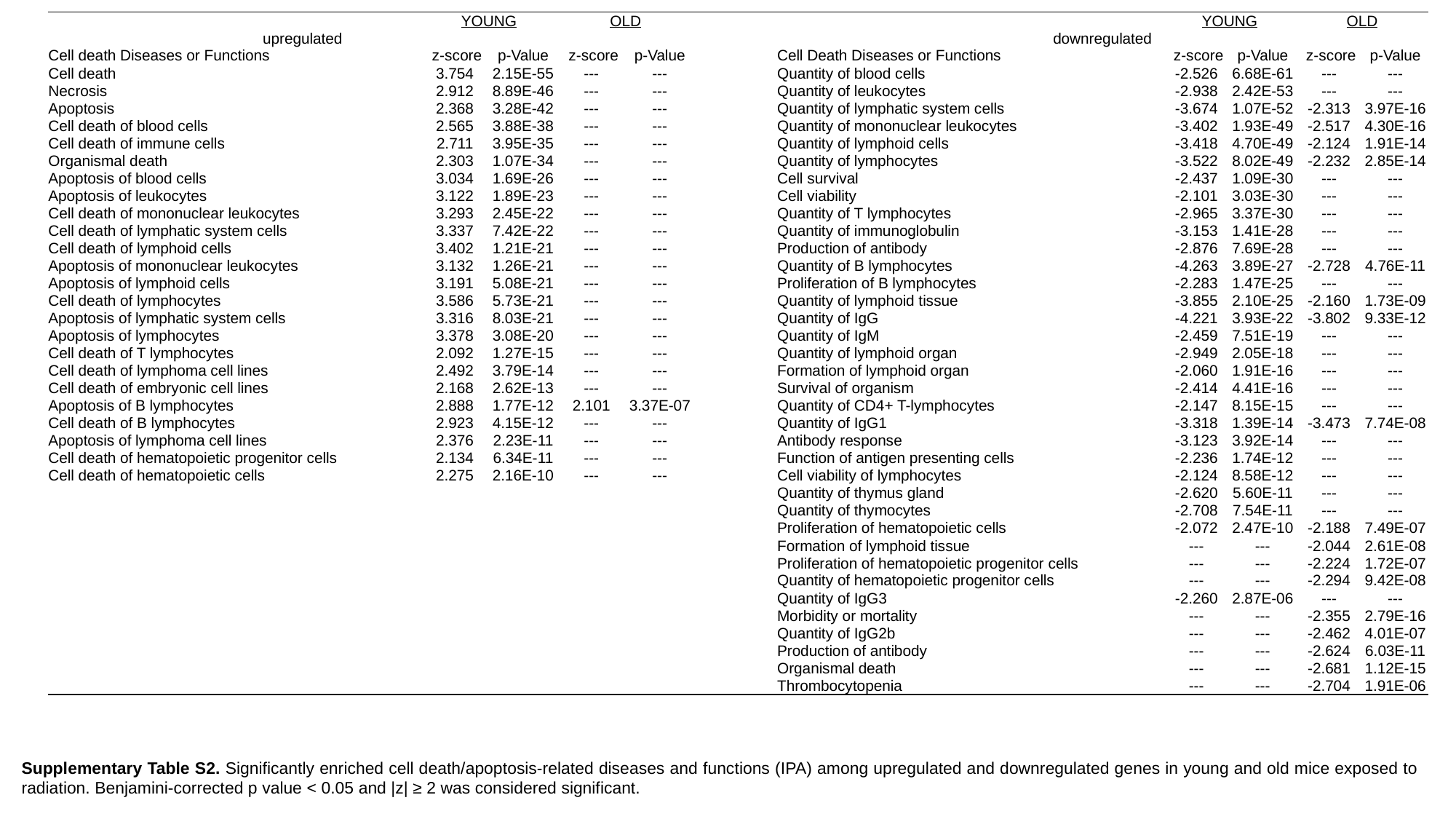

| | YOUNG | | OLD | | | | YOUNG | | OLD | |
| --- | --- | --- | --- | --- | --- | --- | --- | --- | --- | --- |
| upregulated | | | | | | downregulated | | | | |
| Cell death Diseases or Functions | z-score | p-Value | z-score | p-Value | | Cell Death Diseases or Functions | z-score | p-Value | z-score | p-Value |
| Cell death | 3.754 | 2.15E-55 | --- | --- | | Quantity of blood cells | -2.526 | 6.68E-61 | --- | --- |
| Necrosis | 2.912 | 8.89E-46 | --- | --- | | Quantity of leukocytes | -2.938 | 2.42E-53 | --- | --- |
| Apoptosis | 2.368 | 3.28E-42 | --- | --- | | Quantity of lymphatic system cells | -3.674 | 1.07E-52 | -2.313 | 3.97E-16 |
| Cell death of blood cells | 2.565 | 3.88E-38 | --- | --- | | Quantity of mononuclear leukocytes | -3.402 | 1.93E-49 | -2.517 | 4.30E-16 |
| Cell death of immune cells | 2.711 | 3.95E-35 | --- | --- | | Quantity of lymphoid cells | -3.418 | 4.70E-49 | -2.124 | 1.91E-14 |
| Organismal death | 2.303 | 1.07E-34 | --- | --- | | Quantity of lymphocytes | -3.522 | 8.02E-49 | -2.232 | 2.85E-14 |
| Apoptosis of blood cells | 3.034 | 1.69E-26 | --- | --- | | Cell survival | -2.437 | 1.09E-30 | --- | --- |
| Apoptosis of leukocytes | 3.122 | 1.89E-23 | --- | --- | | Cell viability | -2.101 | 3.03E-30 | --- | --- |
| Cell death of mononuclear leukocytes | 3.293 | 2.45E-22 | --- | --- | | Quantity of T lymphocytes | -2.965 | 3.37E-30 | --- | --- |
| Cell death of lymphatic system cells | 3.337 | 7.42E-22 | --- | --- | | Quantity of immunoglobulin | -3.153 | 1.41E-28 | --- | --- |
| Cell death of lymphoid cells | 3.402 | 1.21E-21 | --- | --- | | Production of antibody | -2.876 | 7.69E-28 | --- | --- |
| Apoptosis of mononuclear leukocytes | 3.132 | 1.26E-21 | --- | --- | | Quantity of B lymphocytes | -4.263 | 3.89E-27 | -2.728 | 4.76E-11 |
| Apoptosis of lymphoid cells | 3.191 | 5.08E-21 | --- | --- | | Proliferation of B lymphocytes | -2.283 | 1.47E-25 | --- | --- |
| Cell death of lymphocytes | 3.586 | 5.73E-21 | --- | --- | | Quantity of lymphoid tissue | -3.855 | 2.10E-25 | -2.160 | 1.73E-09 |
| Apoptosis of lymphatic system cells | 3.316 | 8.03E-21 | --- | --- | | Quantity of IgG | -4.221 | 3.93E-22 | -3.802 | 9.33E-12 |
| Apoptosis of lymphocytes | 3.378 | 3.08E-20 | --- | --- | | Quantity of IgM | -2.459 | 7.51E-19 | --- | --- |
| Cell death of T lymphocytes | 2.092 | 1.27E-15 | --- | --- | | Quantity of lymphoid organ | -2.949 | 2.05E-18 | --- | --- |
| Cell death of lymphoma cell lines | 2.492 | 3.79E-14 | --- | --- | | Formation of lymphoid organ | -2.060 | 1.91E-16 | --- | --- |
| Cell death of embryonic cell lines | 2.168 | 2.62E-13 | --- | --- | | Survival of organism | -2.414 | 4.41E-16 | --- | --- |
| Apoptosis of B lymphocytes | 2.888 | 1.77E-12 | 2.101 | 3.37E-07 | | Quantity of CD4+ T-lymphocytes | -2.147 | 8.15E-15 | --- | --- |
| Cell death of B lymphocytes | 2.923 | 4.15E-12 | --- | --- | | Quantity of IgG1 | -3.318 | 1.39E-14 | -3.473 | 7.74E-08 |
| Apoptosis of lymphoma cell lines | 2.376 | 2.23E-11 | --- | --- | | Antibody response | -3.123 | 3.92E-14 | --- | --- |
| Cell death of hematopoietic progenitor cells | 2.134 | 6.34E-11 | --- | --- | | Function of antigen presenting cells | -2.236 | 1.74E-12 | --- | --- |
| Cell death of hematopoietic cells | 2.275 | 2.16E-10 | --- | --- | | Cell viability of lymphocytes | -2.124 | 8.58E-12 | --- | --- |
| | | | | | | Quantity of thymus gland | -2.620 | 5.60E-11 | --- | --- |
| | | | | | | Quantity of thymocytes | -2.708 | 7.54E-11 | --- | --- |
| | | | | | | Proliferation of hematopoietic cells | -2.072 | 2.47E-10 | -2.188 | 7.49E-07 |
| | | | | | | Formation of lymphoid tissue | --- | --- | -2.044 | 2.61E-08 |
| | | | | | | Proliferation of hematopoietic progenitor cells | --- | --- | -2.224 | 1.72E-07 |
| | | | | | | Quantity of hematopoietic progenitor cells | --- | --- | -2.294 | 9.42E-08 |
| | | | | | | Quantity of IgG3 | -2.260 | 2.87E-06 | --- | --- |
| | | | | | | Morbidity or mortality | --- | --- | -2.355 | 2.79E-16 |
| | | | | | | Quantity of IgG2b | --- | --- | -2.462 | 4.01E-07 |
| | | | | | | Production of antibody | --- | --- | -2.624 | 6.03E-11 |
| | | | | | | Organismal death | --- | --- | -2.681 | 1.12E-15 |
| | | | | | | Thrombocytopenia | --- | --- | -2.704 | 1.91E-06 |
Supplementary Table S2. Significantly enriched cell death/apoptosis-related diseases and functions (IPA) among upregulated and downregulated genes in young and old mice exposed to radiation. Benjamini-corrected p value < 0.05 and |z| ≥ 2 was considered significant.

## Slide 4
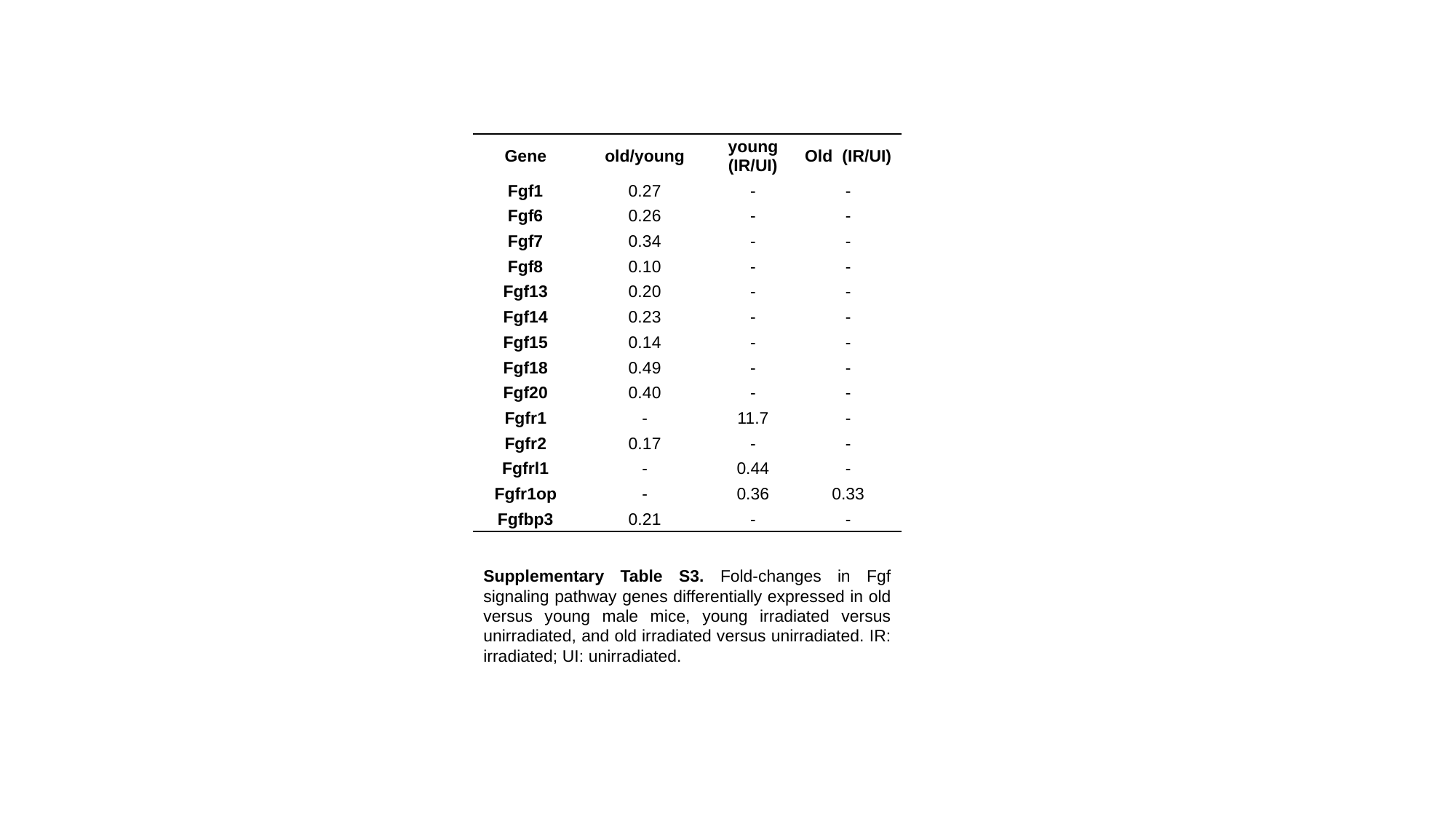

| Gene | old/young | young (IR/UI) | Old (IR/UI) |
| --- | --- | --- | --- |
| Fgf1 | 0.27 | - | - |
| Fgf6 | 0.26 | - | - |
| Fgf7 | 0.34 | - | - |
| Fgf8 | 0.10 | - | - |
| Fgf13 | 0.20 | - | - |
| Fgf14 | 0.23 | - | - |
| Fgf15 | 0.14 | - | - |
| Fgf18 | 0.49 | - | - |
| Fgf20 | 0.40 | - | - |
| Fgfr1 | - | 11.7 | - |
| Fgfr2 | 0.17 | - | - |
| Fgfrl1 | - | 0.44 | - |
| Fgfr1op | - | 0.36 | 0.33 |
| Fgfbp3 | 0.21 | - | - |
Supplementary Table S3. Fold-changes in Fgf signaling pathway genes differentially expressed in old versus young male mice, young irradiated versus unirradiated, and old irradiated versus unirradiated. IR: irradiated; UI: unirradiated.

## Slide 5
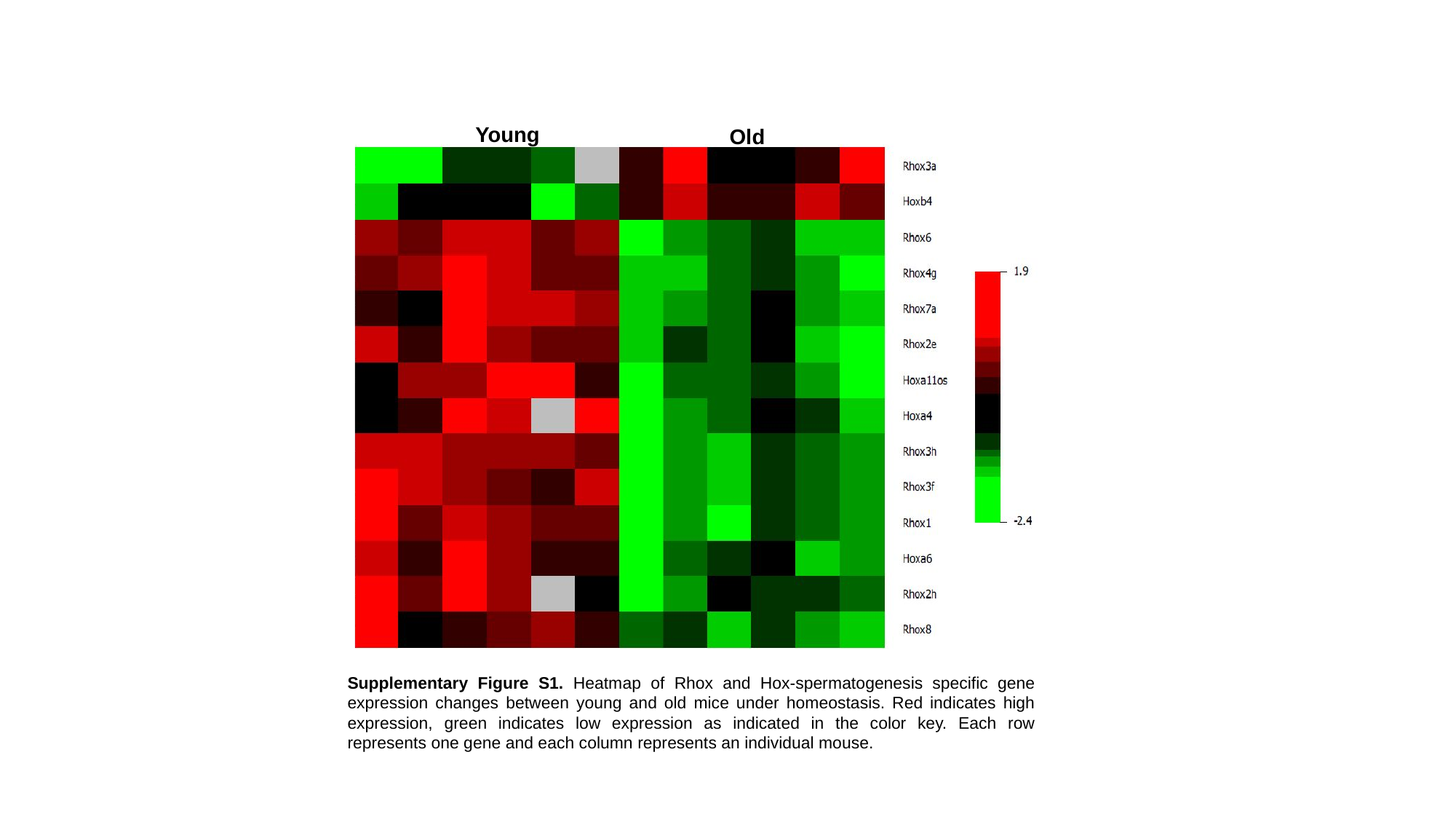

Young
Old
Supplementary Figure S1. Heatmap of Rhox and Hox-spermatogenesis specific gene expression changes between young and old mice under homeostasis. Red indicates high expression, green indicates low expression as indicated in the color key. Each row represents one gene and each column represents an individual mouse.

## Slide 6
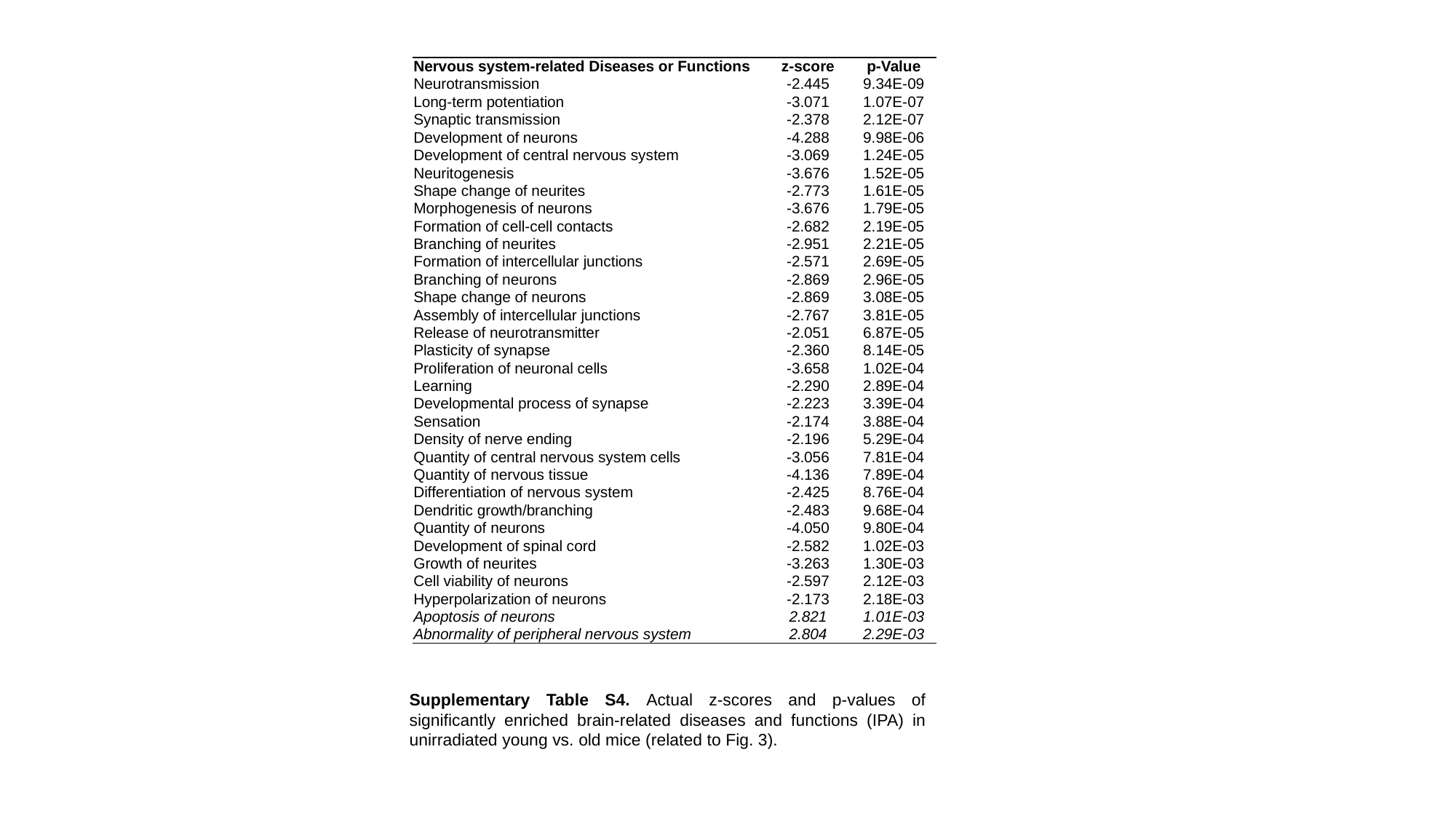

| Nervous system-related Diseases or Functions | z-score | p-Value |
| --- | --- | --- |
| Neurotransmission | -2.445 | 9.34E-09 |
| Long-term potentiation | -3.071 | 1.07E-07 |
| Synaptic transmission | -2.378 | 2.12E-07 |
| Development of neurons | -4.288 | 9.98E-06 |
| Development of central nervous system | -3.069 | 1.24E-05 |
| Neuritogenesis | -3.676 | 1.52E-05 |
| Shape change of neurites | -2.773 | 1.61E-05 |
| Morphogenesis of neurons | -3.676 | 1.79E-05 |
| Formation of cell-cell contacts | -2.682 | 2.19E-05 |
| Branching of neurites | -2.951 | 2.21E-05 |
| Formation of intercellular junctions | -2.571 | 2.69E-05 |
| Branching of neurons | -2.869 | 2.96E-05 |
| Shape change of neurons | -2.869 | 3.08E-05 |
| Assembly of intercellular junctions | -2.767 | 3.81E-05 |
| Release of neurotransmitter | -2.051 | 6.87E-05 |
| Plasticity of synapse | -2.360 | 8.14E-05 |
| Proliferation of neuronal cells | -3.658 | 1.02E-04 |
| Learning | -2.290 | 2.89E-04 |
| Developmental process of synapse | -2.223 | 3.39E-04 |
| Sensation | -2.174 | 3.88E-04 |
| Density of nerve ending | -2.196 | 5.29E-04 |
| Quantity of central nervous system cells | -3.056 | 7.81E-04 |
| Quantity of nervous tissue | -4.136 | 7.89E-04 |
| Differentiation of nervous system | -2.425 | 8.76E-04 |
| Dendritic growth/branching | -2.483 | 9.68E-04 |
| Quantity of neurons | -4.050 | 9.80E-04 |
| Development of spinal cord | -2.582 | 1.02E-03 |
| Growth of neurites | -3.263 | 1.30E-03 |
| Cell viability of neurons | -2.597 | 2.12E-03 |
| Hyperpolarization of neurons | -2.173 | 2.18E-03 |
| Apoptosis of neurons | 2.821 | 1.01E-03 |
| Abnormality of peripheral nervous system | 2.804 | 2.29E-03 |
Supplementary Table S4. Actual z-scores and p-values of significantly enriched brain-related diseases and functions (IPA) in unirradiated young vs. old mice (related to Fig. 3).
